# A Modified Bacillus Calmette-Guérin (BCG) Vaccine with Reduced Activity of Antioxidants and Glutamine Synthetase Exhibits Enhanced Protection of Mice despite Diminished *in Vivo* Persistence

**DOI:** 10.3390/vaccines1010034

**Published:** 2013-01-11

**Authors:** Carolyn M. Shoen, Michelle S. DeStefano, Cynthia C. Hager, Kyi-Toe Tham, Miriam Braunstein, Alexandria D. Allen, Hiriam O. Gates, Michael H. Cynamon, Douglas S. Kernodle

**Affiliations:** 1 Veterans Affairs Medical Center, Syracuse, NY 13212, USA; E-Mails: shoenc@cnyrc.org (C.M.S.); Michelle.Destefano@va.gov (M.S.D.); Michael.Cynamon@va.gov (M.H.C.); 2 Department of Medicine, Vanderbilt University Medical Center, Nashville, TN 37232, USA; E-Mails: cindy.hager@vanderbilt.edu (C.C.H.); Alexandria.Allen@mlh.org (A.D.A.); hirham@bellsouth.net (H.O.G.); 3 Department of Pathology, Vanderbilt University Medical Center, Nashville, TN 37232, USA; E-Mail: kyitoe@catham.org; 4 Veterans Affairs Medical Center, Nashville, TN 37212, USA; 5 Department of Microbiology and Immunology, University of North Carolina, Chapel Hill, NC 27599, USA; E-Mail: braunste@med.unc.edu; 6 Department of Microbiology, Immunology and Pathology, Vanderbilt University Medical Center, Nashville, TN 37232, USA

**Keywords:** tuberculosis, vaccine, Bacillus Calmette-Guérin (BCG), antioxidants, superoxide dismutase, sigma factor, glutamine synthetase, immunity, immune suppression

## Abstract

Early attempts to improve BCG have focused on increasing the expression of prominent antigens and adding recombinant toxins or cytokines to influence antigen presentation. One such modified BCG vaccine candidate has been withdrawn from human clinical trials due to adverse effects. BCG was derived from virulent *Mycobacterium bovis* and retains much of its capacity for suppressing host immune responses. Accordingly, we have used a different strategy for improving BCG based on reducing its immune suppressive capacity. We made four modifications to BCG Tice to produce 4dBCG and compared it to the parent vaccine in C57Bl/6 mice. The modifications included elimination of the oxidative stress sigma factor SigH, elimination of the SecA2 secretion channel, and reductions in the activity of iron co-factored superoxide dismutase and glutamine synthetase. After IV inoculation of 4dBCG, 95% of vaccine bacilli were eradicated from the spleens of mice within 60 days whereas the titer of BCG Tice was not significantly reduced. Subcutaneous vaccination with 4dBCG produced greater protection than vaccination with BCG against dissemination of an aerosolized challenge of *M. tuberculosis* to the spleen at 8 weeks post-challenge. At this time, 4dBCG-vaccinated mice also exhibited altered lung histopathology compared to BCG-vaccinated mice and control mice with less well-developed lymphohistiocytic nodules in the lung parenchyma. At 26 weeks post-challenge, 4dBCG-vaccinated mice but not BCG-vaccinated mice had significantly fewer challenge bacilli in the lungs than control mice. In conclusion, despite reduced persistence in mice a modified BCG vaccine with diminished antioxidants and glutamine synthetase is superior to the parent vaccine in conferring protection against *M. tuberculosis*. The targeting of multiple immune suppressive factors produced by BCG is a promising strategy for simultaneously improving vaccine safety and effectiveness.

## 1. Introduction

Tuberculosis (TB) remains an enormous global health problem despite the vaccination of more than 100 million newborns annually with Bacillus Calmette-Guérin (BCG), the current live-attenuated vaccine against TB [1,2]. The high prevalence of HIV infection in some countries combined with the rising incidence of infection caused by extensively drug-resistant strains of *Mycobacterium tuberculosis* threaten to make a dire global TB situation even worse.

BCG has been used as a vaccine against TB for 9 decades. It was identified in 1921 as an attenuated mutant of *M. bovis* and was cultivated for decades in laboratories throughout the world. During this time it underwent divergent evolution and the currently-available BCG daughter strains (substrains) differ from each other and from the original BCG vaccine, which no longer exists [3,4]. BCG provides 80% protection against miliary and meningeal TB in childhood and the routine vaccination of newborns in much of the world is estimated to prevent about 40,000 cases annually [5,6]. In early studies, BCG was also highly efficacious against pulmonary TB with about 80% protection over the first 2 decades and up to 50% protection was still evident 6 decades after vaccination [7,8,9]. However, the effectiveness of the BCG daughter strains against pulmonary TB appears to have declined over time for reasons that continue to be debated [10,11]. Pulmonary TB is much more common than the disseminated forms of TB and accounts for the vast majority of the TB global burden. Pulmonary TB is also the contagious form of TB and thus an effective vaccine against pulmonary TB should reduce all forms of TB.

In an effort to restore BCG’s ability to protect against pulmonary TB, we have enhanced BCG’s immunogenicity by reducing the activity and secretion of microbial antioxidants [12]. This approach differs markedly from the more common strategy of modifying BCG by increasing its production of prominent antigens and adding recombinant toxins or cytokines [13,14,15]. However our approach is well-founded in the context of reports that mycobacterial antioxidants suppress host immune responses and there is growing evidence that they also promote the pathogenesis of granulomatous inflammation [16,17,18,19,20,21,22]. Furthermore, it appears that as BCG was cultivated *in vitro* for decades it increased its production of antioxidants by a process involving duplication of regions of chromosomal DNA and other mutations [4], and this increase may partly explain the decline in its protective efficacy against pulmonary TB over time [11,12]. Thus, although we have not specifically undone the multiple mutations that arose in BCG during decades of *in vitro* cultivation, reducing the activity of microbial antioxidants makes our modified BCG more like the early BCG vaccine that was effective in preventing pulmonary TB.

In the present investigation we make four modifications to BCG including reducing the activity of glutamine synthetase (GlnA1), a secreted mycobacterial enzyme implicated in immune evasion [23,24,25], thereby producing 4dBCG with diminished antioxidant and GlnA1 activity. We then compare 4dBCG to BCG *in vivo* including an assessment of the persistence of vaccine strains in the spleens of mice. We also use a vaccination-challenge model to assess protection against hematogenous dissemination and lung pathology after aerosol inoculation of vaccinated mice with *M. tuberculosis*. We find that although 4dBCG is cleared more rapidly than BCG from the spleens of mice, it provides greater protection against dissemination of an aerosolized challenge with *M. tuberculosis* and also alters lung histopathology. The clinical implications of these findings are discussed in the context of the difficulties in correlating results in mice with results in man.

## 2. Results and Discussion

### 2.1. Construction of 4dBCG

**Figure 1:**
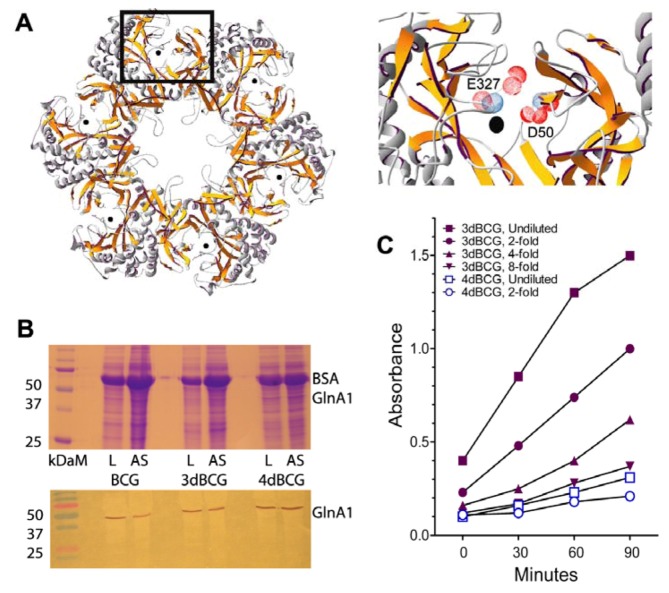
Construction of 4dBCG. (**A**) Hexameric ring representing half of the enzymatically-active dodecameric form of GlnA1 (left) and enlargement (right) of the area within the rectangle to show the deleted amino acids in relationship to the active site manganese atom, represented by the black dot, with amino acids numbered according to the glutamine synthetase of *Salmonella* [30]. As the active site comprises residues from adjacent monomers, insertion of one ∆D50∆E327 dnGlnA1 monomer into the ring is predicted to inactivate two active sites. (**B**) SDS-PAGE (upper) and immunoblot (lower) for GlnA1 of lysates of Bacillus Calmette-Guérin (BCG), 3dBCG and 4dBCG. Lanes are labeled on the figures as L (lysate) and AS (ammonium sulfate-treated lysate) for each strain, kDaM = Kilodalton markers. (**C**) Graph of a representative experiment comparing the glutamine synthetase activity of undiluted and diluted AS preparations from 3dBCG and 4dBCG as determined by monitoring A_540_ over time.

Details of the construction of 3dBCG, with three genetic modifications of the parent BCG Tice vaccine strain, have been previously reported [12]. Two of the modifications involved allelic inactivation of *sigH*, the oxidative stress sigma factor [20,26,27] and *secA2*, the secretion channel for iron co-factored superoxide dismutase (SodA) [17,18]. A third modification involved recombinant expression of a dominant-negative ∆H28∆H76 mutant of *sodA* that reduced SodA activity by more than 90% compared to the parent vaccine. GlnA1 is another microbial factor implicated in immune suppression that has been shown to confer resistance to killing of *M. tuberculosis* by human macrophages [24,25]. As GlnA1 is essential for the growth of *M. tuberculosis* [28] we partially lowered GlnA1 activity by using dominant-negative interference techniques while preserving potentially important epitopes for immune recognition [29]. First we deleted two amino acids that are important for enzymatic activity, an aspartic acid at position 50 and glutamic acid at position 327 (Figure 1A). Then we inserted the allele encoding the ∆D50∆E327 GlnA1 monomer on a plasmid vector into 3dBCG to yield 4dBCG. Immunoblotting demonstrated that enzyme activity was reduced 8-fold in 4dBCG, verifying a dominant-negative effect (Figure 1B,C).

### 2.2. 4dBCG Is Cleared More Rapidly than BCG from the Spleens of Mice

BCG daughter strains that exhibit relative invasiveness and persistence in animal models such as BCG Pasteur 1173P2 and BCG Danish 1331 cause more adverse effects in man than less virulent BCG substrains such as BCG Tokyo 172 [1,31,32]. In the context of the recent withdrawal of the recombinant BCG vaccine candidate Aeras-422 from human clinical trials due to adverse effects [33,34], assessment of the virulence of the live vaccine strains has become an increasingly important part of the initial evaluation of a live vaccine candidate.

**Figure 2:**
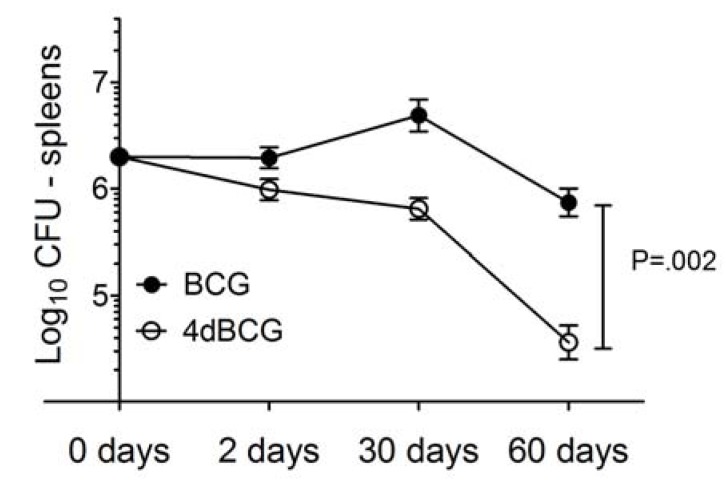
Spleen titers of BCG and 4dBCG in C57Bl/6 mice. Mean ± SEM spleen titers on day 2, day 30, and day 60 after IV inoculation of mice with 2 × 10^7^ cfu of BCG or 4dBCG. Each value represents four to six mice. The P value represents the comparison of the BCG and 4dBCG groups at 60 days. The day 0 value is estimated as 10% of the original IV inoculum.

To determine whether or not our modifications altered the *in vivo* persistence of vaccine bacilli, we administered 2 × 10^7^ cfu of BCG or 4dBCG intravenously to C57Bl/6 mice. At day 60 post-inoculation the mean titer of BCG was lower, albeit not significantly, than the mean titer at day 2 post-inoculation *p *= 0.07, Figure 2). In contrast, the number of 4dBCG bacilli fell by more than 95% over the same period of time (*p* = 0.01) and was 20-fold less than the titer of BCG at day 60 post-inoculation (*p* = 0.002). In summary, 4dBCG did not persist as well as the parent BCG vaccine in the spleens of mice. The reduced persistence of 4dBCG may partly reflect a slower intrinsic growth rate combined with greater unmasking and activation of innate and adaptive host responses that mediate the clearance of live mycobacteria from host organs.

### 2.3. Vaccination with 4dBCG Exhibits Greater Protection than BCG against Early Dissemination of Aerosolized M. tuberculosis to the Spleen

For nearly 6 decades it has been appreciated that BCG substrains differ in their ability to interfere with the hematogenous dissemination of a challenge dose of *M. tuberculosis* in animal models [35]. Furthermore, relatively virulent BCG substrains capable of persisting in relatively high titer in the organs of mice protect best in small animal models and thus have been considered by some authorities to have greater vaccine potency [35,36,37,38,39,40,41]. Accordingly, we wondered whether our modifications to BCG that reduced the *in vivo* persistence of 4dBCG would also reduce the ability of 4dBCG to protect against hematogenous dissemination.

**Figure 3:**
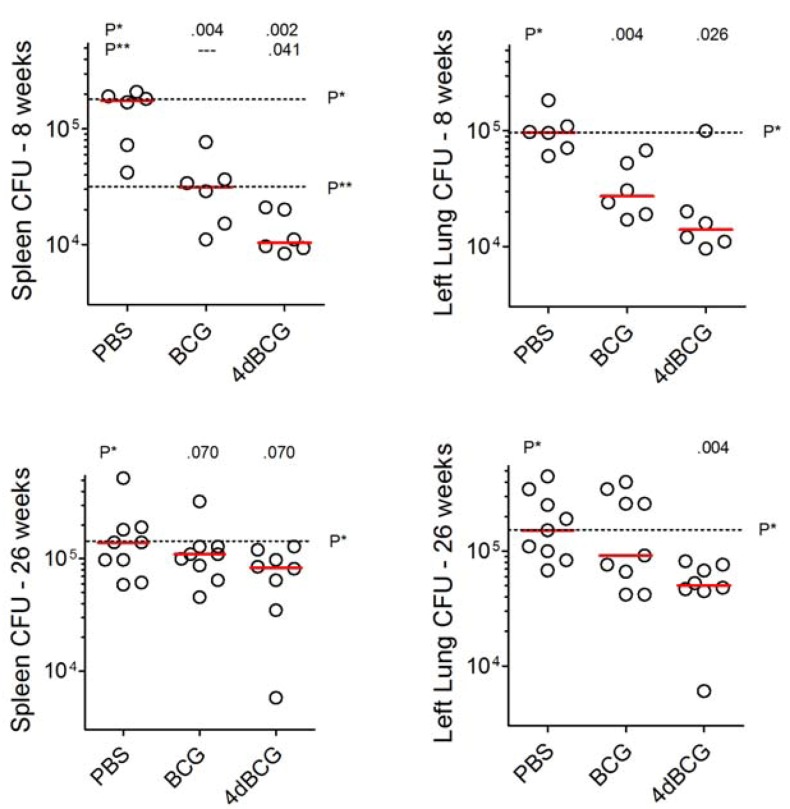
Titer of *M. tuberculosis* challenge bacilli in the spleens and left lungs of C57Bl/6 mice. Mice received aerosol challenge with 100 cfu of *M. tuberculosis* strain Erdman S-1. The whole spleens and left lungs were assessed for cfu titer while the right lungs were processed for histopathology as shown in Figure 4, Figure 5, Figure 6 and Figure 7. The individual data points (circles) and median (bar) for each vaccination arm are shown at 8 weeks (top, six mice per vaccination arm) and 26 weeks (bottom, eight or nine mice per vaccination arm) post-challenge. The dotted lines extend the bar representing the median value of the PBS-vaccinated and BCG-vaccinated groups and are marked P* and P**, respectively. Analysis of spleen and lung results at 8 weeks and lung results at 26 weeks by 1 way ANOVA demonstrated that the median values of the groups varied significantly by Kruskal-Wallis test. The groups were also compared using the Mann-Whitney test and values in the row labeled P* above the panel indicate the comparison of each vaccinated group against the phosphate-buffered saline (PBS) group. The values in the row labeled P** indicate the comparison of the 4dBCG and BCG groups.

We compared mice vaccinated with BCG or 4dBCG to control mice that were vaccinated with phosphate-buffered saline (PBS), using 15 mice per vaccination arm. At 8 weeks after subcutaneous vaccination with 2 × 10^7^ cfu of the vaccine strains, mice were challenged by aerosol with 100 cfu of *M. tuberculosis* strain Erdman S-1. To assess hematogenous dissemination, we determined spleen cfu counts at 8 weeks post-aerosol challenge. At this time, PBS-vaccinated mice harbored a median of 1.8 × 10^5^ bacilli in the spleen. CFU titers were 5.8-fold lower in BCG-vaccinated mice (median, 3.1 × 10^4^ cfu) and 18-fold lower in 4dBCG-vaccinated mice (median, 1.0 × 10^4^ cfu, Figure 3).

Lung cfu titers were also determined. PBS-vaccinated mice exhibited a median of 9.7 × 10^4^ cfu at 8 weeks post-challenge and titers were 3.6-fold lower in BCG-vaccinated mice (median, 2.7 × 10^4^ cfu) and 7.5-fold lower in 4dBCG-vaccinated mice (median, 1.3 × 10^4^ cfu). These results paralleled the findings in the spleen and represent the growth over time of the original aerosolized inoculums of bacteria within the lung combined with bronchogenic and hematogenous dissemination to other parts of the lung. Results at 26 weeks are described below (Section 2.5)

### 2.4. Vaccination with 4dBCG Alters the Pattern of Lymphohistiocytic Infiltration of Lung Parenchyma at 8 Weeks Post-Challenge

**Figure 4:**
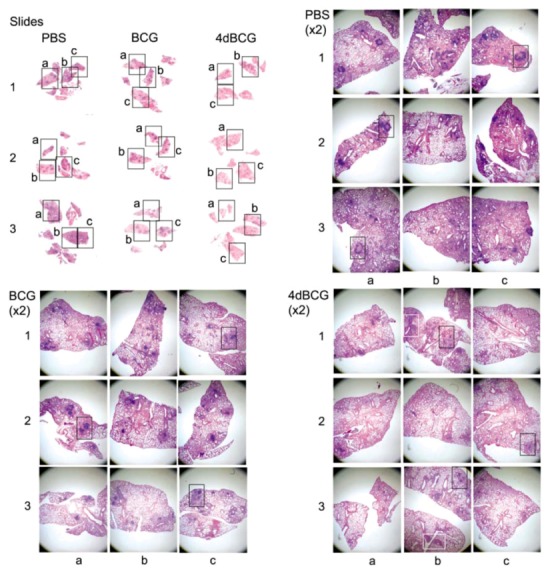
Low-power photomicrographs of sections of lung tissue. (Upper left panel, labeled “Slides”) Photographs of the microscope slides containing H&E-stained lung sections are shown from right lungs of three mice from each of the PBS, BCG, and 4dBCG vaccination arms at 8 weeks post-aerosol challenge with 100 cfu of *M. tuberculosis* strain Erdman S-1. Three rectangles are labeled a, b, and c and cover approximately 70–80% of the lung tissue on each slide. (Panels labeled “PBS”, “BCG”, and “4dBCG”) Three low-power photomicrographs of the lung tissue were taken through the 2× microscope objective and correspond to the three rectangles in each lung labeled a, b, and c in the “slides” panel. Within these photomicrographs, the black rectangles highlight lymphohistiocytic nodules in relatively consolidated regions of lung tissue, which are enlarged further and displayed in Figure 5. The white rectangles in the 4dBCG group of mice outline lung tissue containing bronchus-associated lymphoid tissue (BALT) and are displayed in Figure 7.

CFU counts are an incomplete measure of vaccine-induced protection against pulmonary TB, and thus we also evaluated the effect of vaccination upon lung pathology. To display these results objectively, we took low power photomicrographs (×2 magnification) of H&E-stained sections of lung tissue covering about 70% of the lung tissue on each slide for three mice per vaccination arm (Figure 4). We marked the parts of the low-power photomicrographs that were enlarged further to display representative histopathologic features. Thus the reader can visualize the representative features in the context of the whole lung cross-sections. As shown in Figure 4, regions of relatively unconsolidated lung parenchyma were observed in all three vaccination arms. Mice vaccinated with PBS exhibited relatively more inflammation and consolidation of lung tissue at 8 weeks post-aerosol challenge with *M. tuberculosis* compared to mice vaccinated with BCG and mice vaccinated with 4dBCG.

**Figure 5:**
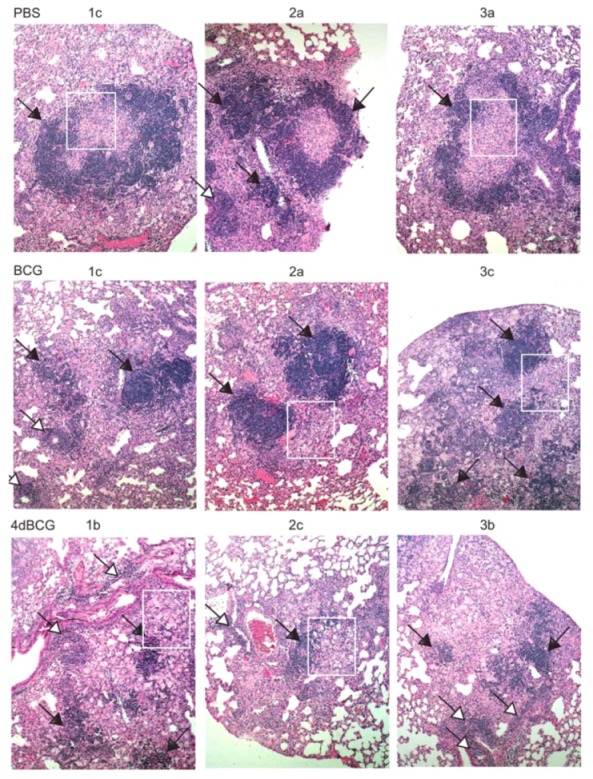
Lymphohistiocytic nodules in the lung parenchyma. Mid-power (×10 microscope objective) photomicrographs of regions of lung consolidation within the tissue sections in Figure 4 are displayed at a greater magnification. Most of the areas that stain dark blue in the PBS and BCG groups represent dense collections of lymphocytes within the lung parenchyma that are accompanied by lymphohistiocytic infiltration and consolidation of the alveolar spaces. Examples of these regions are indicated with arrows (black arrowheads) and the white rectangles outline regions of lung tissue that are enlarged further in Figure 6. Lymphoid aggregates were also observed in the lung parenchyma in the 4dBCG group of mice however they were typically smaller with less dense alveolar infiltrate compared to the PBS and BCG groups. The arrows with white arrowheads indicate BALT, which is distinguished from lymphohistiocytic nodules in the lung parenchyma by their association with blood vessels and bronchi.

**Figure 6:**
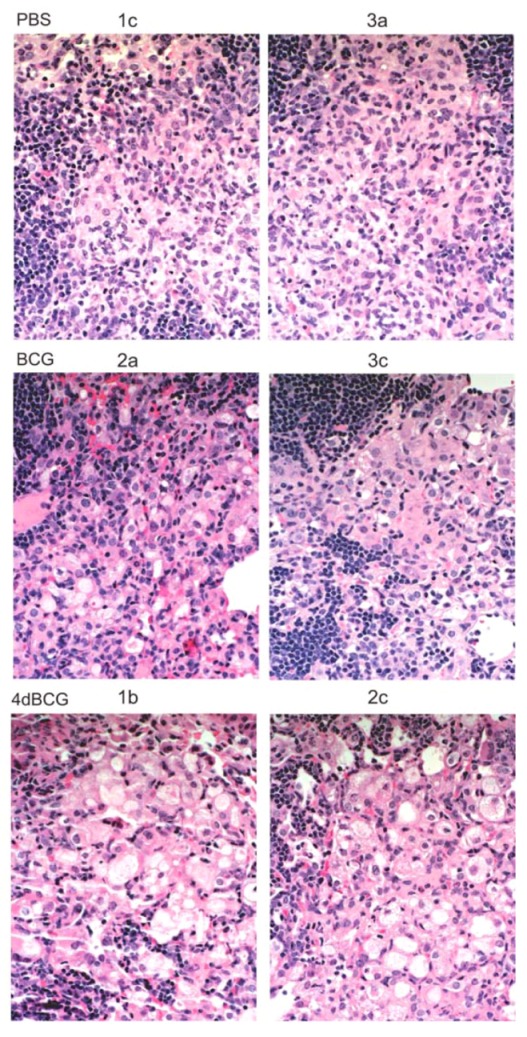
High-power views of lymphohistiocytic nodules. Consolidated lung tissue with many epithelioid cells containing pale oval nuclei and eosinophilic cytoplasm are observed in the PBS-vaccinated mice and to a lesser degree in the BCG-vaccinated mice. The alveolar spaces adjacent to the smaller lymphoid nodules in the 4dBCG-vaccinated mice are edematous, contain relatively fewer epithelioid cells, and exhibit greater preservation of alveolar wall architecture than in the PBS- and BCG-vaccinated mice.

**Figure 7:**
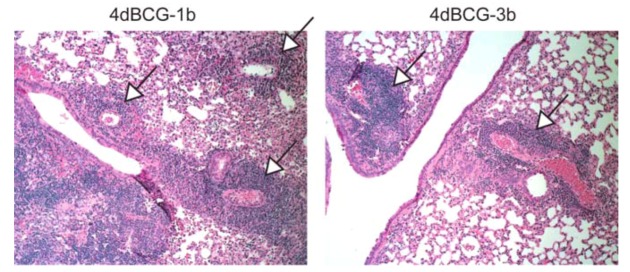
Bronchus-associated lymphoid tissue (BALT). The panels display ×10 enlargements of the lung sections from mice vaccinated with 4dBCG as represented by the white rectangles in Figure 4. BALT was prominent along the central bronchovascular structures in all three groups of mice (see Figure 5) and its association with blood vessels and bronchi distinguishes BALT from collections of lymphocytes in the lung parenchyma.

The most apparent difference in lung histopathology between vaccination arms involved the lymphohistiocytic nodules, which stain dark blue due to the density of nuclei and scant cytoplasm within lymphocytes (Figure 4 and Figure 5). They were most numerous and well-developed in the PBS vaccination arm, often exhibiting a circumferential or near-circumferential ring of lymphocytes around epithelioid cells that had replaced normal alveolar architecture (Figure 5 and Figure 6). Also staining dark blue was bronchus-associated lymphoid tissue (BALT). BALT typically can be distinguished from lymphohistiocytic nodules within the lung parenchyma by its association with bronchovascular structures (Figure 5 and Figure 7) and was observed in all 3 vaccination arms. 

Compared to BCG, the lungs of mice vaccinated with 4dBCG exhibited smaller collections of lymphocytes within the lung parenchyma with less disruption of alveolar architecture as demonstrated by better preservation of the alveolar walls and fewer epithelioid cells (Figure 5 and Figure 6). There was also more generalized eosinophilic staining of the lungs of mice vaccinated with 4dBCG (Figrue 4), due in part to extravasated RBCs and edema fluid.

### 2.5. Vaccination with 4dBCG Reduces Lung Cfu Counts at 26 Weeks Post-Challenge

Due to weight loss in PBS-vaccinated mice the experiment was limited to 26 weeks post-challenge. At this time the mice vaccinated with BCG or 4dBCG exhibited spleen cfu titers that were not significantly lower than cfu titers in the PBS vaccination arm (Figure 3). Lung histopathology revealed extensive granulomatous inflammation in all 3 groups of mice (not shown). Lung cfu titers in the 4dBCG vaccination arm were significantly lower than titers in the PBS vaccination arm (median values of 5.0 × 10^4^ cfu and 1.5 × 10^5^ cfu, respectively, *p* = 0.004).

### 2.6. Advantages and Limitations of This Vaccine Strategy and Its Implications for Human Vaccination

The results of these experiments support efforts to improve the safety and effectiveness of BCG by targeting microbial factors that suppress host immune responses. We previously have reported that 3dBCG, with 3 attenuating modifications, surpasses the parent BCG Tice vaccine in the induction of primary and secondary immune responses and in the clearance of challenge bacilli in a memory-immune model of subcutaneous vaccination followed by intravenous challenge [12]. Other groups of TB vaccine investigators have reported that the immunogenicity and protective efficacy of BCG can be improved by disrupting *sapM* and *zmp1*, microbial genes that encode, respectively, an acid phosphatase and zinc metalloprotease implicated in inhibition of phagosome maturation [42,43,44,45]. Collectively, there is now substantial evidence that the immunogenicity of BCG can be improved by targeting immune suppressive microbial factors.

The present investigation further explores this strategy for improving BCG and demonstrates that despite diminished persistence *in vivo* 4dBCG surpasses the parent BCG Tice vaccine in protecting mice against the dissemination of an aerosolized challenge of *M. tuberculosis* from the lung to the spleen. Vaccination with 4dBCG also altered lung histopathology to result in fewer and less well-developed lymphohistiocytic nodules within the lung parenchyma at 8 weeks post-challenge. At 26 weeks post-challenge mice vaccinated with 4dBCG had significantly fewer bacilli in the lungs than control mice. The biological mechanisms that underlie the improved protection observed with 4dBCG have not been fully explored, however oxidants and glutamate are signaling molecules that activate antigen-presenting cells and T cells [46,47,48,49,50], promote apoptosis and apoptosis-associated cross presentation of bacterial antigens to induce CD8+ T cell responses [49,51,52], and influence the balance between Th1 and Th17 responses [53,54]. Thus our modifications to BCG may have enhanced protection by unmasking protective host responses that are suppressed by the parent BCG vaccine.

There are several issues worthy of discussion regarding how these results fit into the TB vaccine literature and their implications for human vaccination. First, it is noteworthy that we used a relatively high dose of 4dBCG and BCG, 2 × 10^7^ cfu, which is greater than the 3.7 × 10^4^ cfu to 3 × 10^6^ cfu typically used for human vaccination [1]. The ability of some BCG substrains to induce protection with doses as low as 10^1^ cfu has been interpreted by some experts as indicative of vaccine potency [32,55,56,57,58] such that vaccines with this capability including Pasteur 1173P2 and Danish 1331 are called “strong” strains whereas those without this capability including Tokyo 172 are called “weak” [59,60]. The biological reason for this difference has traditionally been attributed to differences in the ability of the live vaccine to multiply within the host and the BCG vaccines exhibiting the smallest allergenic, *i.e*., DTH-inducing, doses also exhibit greater lethality in golden hamsters and cause more adverse effects in man [32,59,60,61,62]. As 4dBCG was less capable of persisting in the spleens of mice than the parent BCG Tice vaccine it behaves more like the “weak” BCG daughter strains and accordingly we used a relatively high dose for vaccination. It is possible that a smaller dose of 4dBCG would not confer as much protection. Despite this uncertainty, we have concerns about the ability of some BCG substrains to induce protection with a low dose; in the context of a modern understanding of host-pathogen interactions this capability indicates that the vaccine strain is able to suppress the host responses needed to eradicate live mycobacteria, which is not a good property for a vaccine.

A second issue is that the protection induced by vaccination with 4dBCG waned over time. This was not unexpected as the waning of protection is characteristic of vaccination with BCG in small animal models [63]. Recent results involving C57Bl/6 mice and the same lot of the Erdman challenge strain used in our study found that vaccination with BCG Pasteur conferred protection at 4 and 10 weeks post-aerosol challenge, but not at 20 weeks [64]. Thus, although the magnitude of protection induced by BCG and 4dBCG in our study declined over time, these results are consistent with prior experience and our observation of continued, albeit reduced, protection at 26 weeks post-challenge in the 4dBCG vaccination arm exceeds the duration of protection usually observed in this model. Extrapolation from such results to expectations regarding vaccine effectiveness in man is difficult however it is important to remember that whereas C57Bl/6 mice invariably develop progressive infection that damages the lung [65] 90% of human hosts control aerogenic infection with *M. tuberculosis* for the duration of their lives. Accordingly, the real goal of vaccination against *M. tuberculosis* is to induce in the 10% of persons who are relatively susceptible to the development of active TB the type(s) of immune responses that protect 90% of us. As the immune system of man exhibits important differences from the immune system of mice [66] and is superior in restricting the growth of mycobacteria *in vivo* [21,67], it is possible that the modest enhancements in protection we observed in mice may correlate with enhanced protection against pulmonary TB in man.

A third issue is that although vaccination altered lung histopathology and 4dBCG induced greater changes than BCG in comparison with control mice, the implications of these findings are unclear. In C57Bl/6 mice, the dense aggregates of lymphocytes in the lung parenchyma are comprised primary of B cells and surrounded by macrophages [68,69]. Defects in CXCL13 and IL-23 signaling have been associated with fewer and smaller B cell follicles in mice along with impaired containment of *M. tuberculosis* [70], suggesting that the follicles contribute to protection. Yet most human hosts are able to resolve the granulomatous lesions of primary infection with *M. tuberculosis*, which are often marked by foci of calcification within the lung parenchyma and hilar lymph nodes to form a Ghon complex [71]. Thus the presence of fewer and smaller lymphohistiocytic nodules in 4dBCG-vaccinated mice could represent a defect in the formation of these structures or instead reflect the unmasking of host responses involved in resolving granulomatous inflammation. There is growing appreciation in man that immune surveillance by CD8+ T cells helps to prevent latent TB infection from developing into active pulmonary TB [72,73,74]. Although such responses are not expressed well enough in mice to prevent eventual lung destruction, they may tip the balance towards the resolution of granulomatous inflammation and the containment of infection in human hosts who are intrinsically better than mice at controlling *M. tuberculosis*.

**Figure 8:**
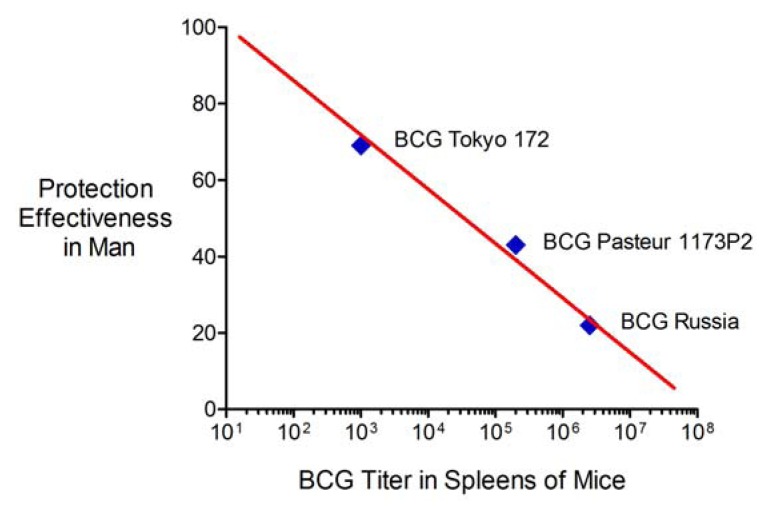
Inverse correlation between protection effectiveness in man and spleen titer in mice of three BCG daughter strains. The protection effectiveness values were taken from a cohort study in Kazakhstan [80] and involved BCG Russia (Microgen), Pasteur 1173P2 (Serbian “Torlak” vaccine), and BCG Tokyo 172 (Japan BCG Laboratory). The log10 spleen titer values were taken from a comparison of 5 BCG strains in Balb/C mice [37] and represent the values at 12 weeks after intravenous inoculation of mice with 10^6^ cfu of the vaccine strain.

The fourth issue is a question that is at the heart of any attempt to improve BCG. Is it better to administer a relatively virulent live vaccine that persists *in vivo* such that immune effector cells are already present when the host subsequently becomes infected with *M. tuberculosis* or instead is it better to use a less immune suppressive vaccine that induces host immune responses that not only eradicate the vaccine strain but also produce memory immunity that can be recalled years later when the host becomes infected? The current BCG daughter strains provide some insight into this important question. More than 40 years ago, two relatively “strong” BCG daughter strains, Pasteur 1173P2 and Danish 1331, were selected for evaluation in a large randomized prospective clinical trial of BCG vaccination in India on the basis of their relative ranking in animal models together with concern about the possible over-attenuation of BCG [32,58,60,61,75]. Yet neither vaccine was effective [76]. Although this was the only randomized trial to compare the protective efficacy of different BCG vaccines, it has subsequently been suggested that case-control studies in which a BCG substrain is replaced by another BCG substrain during the study interval can provide insight into their relative effectiveness. This type of analysis demonstrates that the relatively “weak” BCG Tokyo 172 vaccine confers 50–60% protection against pulmonary TB and is more effective in man than the “strong” Pasteur 1173P2 and Danish 1331 vaccines [77,78,79]. A recent cohort study in Kazakhstan reported similar findings with BCG Tokyo 172 exhibiting greater protection effectiveness than BCG Russia and a Serbian formulation of BCG Pasteur 1173P2 [80]. Correlating these results in man with the results from an earlier report of the persistence of the same BCG substrains in mice [37] answers the question posed at the beginning of this paragraph. In effect, there is an inverse correlation between the protection effectiveness of a BCG substrain in man and its persistence in the spleens of mice (Figure 8). This important observation further justifies efforts to improve BCG by reducing its capacity for immune suppression. It also invites a reevaluation of the historical criteria used to evaluate the potency of BCG daughter strains and to designate them as “strong” or “weak”.

Finally, the inverse correlation noted above raises a fifth issue about our study. In essence, Figure 8 suggests that the BCG substrain used for genetic modifications will be an extremely important variable in vaccine effectiveness. We used BCG Tice, a vaccine strain that historically was considered to be of relatively low virulence [36] but was reformulated with a subculture of BCG obtained from the Institut Pasteur in 1951 and subsequently has exhibited *in vivo* persistence in small animal models similar to that observed with BCG Pasteur 1173P2 [81,82,83]. Our modifications to BCG Tice reduced *in vivo* persistence and per the inverse correlation shown in Figure 8 we expect that 4dBCG will exhibit enhanced protection effectiveness in man. However, we can only speculate as to the magnitude of the improvement and whether 4dBCG will be more or less protective in man than BCG Tokyo 172. Although our modifications were a step in the right direction, ideally the starting strain used for genetic modifications should be a BCG substrain with high protection effectiveness in man. Some progress towards this goal has been achieved; in 2009, while the Aeras Global TB Vaccine Foundation held an exclusive license for Vanderbilt University’s pro-apoptotic bacterial vaccine technology [84], and based in part on our concern about the potential triplication of *sigH* and other virulence genes in recombinant vaccines derived from BCG Danish 1331 [85] that Aeras was using for vaccine construction [15], we encouraged Aeras to instead introduce the genetic modifications described in the current study and our earlier work [12] into BCG Tokyo 172. Two of these modifications, inactivation of SecA2 and the chromosomal insertion of a dominant-negative ΔH28ΔH76 *sodA* allele, were introduced into a GMP (good manufacturing practices) stock of BCG Tokyo 172 (Final Progress Report from Aeras to Vanderbilt University, May 2011). Although Aeras decided not to develop this vaccine candidate or modify it further, based on the findings of this study and the experience with “weak” and “strong” BCG vaccines in man (Figure 8), we expect that it would likely exhibit enhanced protection against pulmonary TB.

In summary, we have described an alternative strategy for modifying BCG based on reducing the activity and secretion of multiple immune suppressive microbial factors. Our vaccine strategy challenges the traditional prioritization of BCG vaccines based on their performance in small animal models. Indeed, we consider the virulence attributes of the “strong” BCG vaccines that have previously been regarded as indicators of potency to instead reflect their immune suppressive capacity. In the context of the repeated failure of “strong” BCG vaccines to exhibit protection against pulmonary TB in man and their associated adverse effects, it is time to consider a different direction in TB vaccine development based on the construction and evaluation of modified BCG vaccines with diminished immune suppressive capacity.

## 3. Experimental

### 3.1. Bacterial Isolates, Plasmids, Antibiotics, and Media

Genetic tools and bacterial isolates are listed in Table 1. *E. coli* strains were grown in LB media. Genetic modifications were introduced into BCG Tice. To prepare vaccine inocula BCG Tice and 4dBCG were grown in Middlebrook 7H9 media with 10% oleic acid-dextrose-catalase (OADC) enrichment supplemented with 0.2% glycerol, and 0.05% Tween80. Kanamycin (50 μg/mL or 25 μg/mL), apramycin (50 μg/mL), and hygromycin B (100 μg/mL or 50 μg/mL) were used to select colonies after genetic manipulations in *E. coli* or BCG, respectively. Erdman S-1 (lot K01) was provided by the Center for Biologics Evaluation and Research, Food and Drug Administration (FDA), USA in accordance with a collaborative agreement between the FDA and the World Health Organization. This challenge strain is a standardized preparation made available to TB vaccine investigators to help reduce variability between laboratories and in two recent studies that compared several *M. tuberculosis* challenge strains, Erdman S-1 demonstrated relatively high capacity for disseminating to the spleen after aerosol challenge [61,86]. 

**Table 1:** Tools for genetic manipulations and bacterial strains.

Name	Description	Reference or source
**Plasmids**		
pCR2.1-TOPO	Plasmid for cloning PCR products	Invitrogen Corp., Carlsbad, California
pBC SK+	*E. coli* phagemid vector	Stratagene, La Jolla, CA
pHV203	*E. coli*-mycobacterial shuttle plasmid with kanamycin resistance gene	[16]
pHV203-dn*glnA1*-ΔD54ΔE335	pHV203 with ΔD54ΔE335 dn mutant *glnA1 *gene with its own promoter	This study
**Strains**		
Erdman S-1	*M. tuberculosis* WHO Standard lot 1 (Mtb S-1) prepared from virulent Erdman lot K01 from the Trudeau Institute and filled by Mycos Research, Loveland, CO	Amy Yang, FDA, Bethesda, MD [64]
TOP 10	Host strain for cloning PCR products, used in combination with pCR2.1-TOPO	Invitrogen Corp., Carlsbad, California
DH5α	*E. coli* host strain for genetic manipulation, construction of mutant enzyme expression vectors	Life Technologies, Gaithersburg, MD [87]
BCG Tice	Bacillus Calmette-Guérin, substrain Tice	Organon Teknika Corp., Durham, NC [82,83]
**3rd generation modified BCG vaccine (three modifications)**		
3dBCG	BCG Tice with inactivated *sigH* and *secA2* containing the chromosomal integration vector pMP399-dn*sodA*-ΔH28ΔH76	[12]
**4th generation modified BCG vaccine (four modifications)**		
4dBCG	BCG Tice with inactivated *sigH* and *secA2* containing pMP399-dn*sodA*-ΔH28ΔH76 and pHV203-dn*glnA1*-ΔD54ΔE335	This study

### 3.2. Molecular Genetic Manipulations

Plasmid and chromosomal integration vectors were electroporated as previously described [12,88]. After electroporation, Middlebrook 7H9 media was added to the samples, which were incubated in 5% CO_2_ at 37 °C for 24 h before the suspension was plated on Middlebrook 7H11 agar containing antibiotics as needed. Successful transformation was confirmed by PCR of DNA unique to the vector.

To construct dominant-negative (dn) enzyme monomers, *glnA1* was PCR-amplified from DNA of *M. tuberculosis* strain H37RV, ligated into pCR2.1-TOPO, and propagated in *E. coli* TOP 10. Site-directed mutagenesis was performed using primer overlap extension methods [89]. Codon deletion was verified by DNA sequencing and the sequence data was deposited in GenBank, Accession No. HM217184.

### 3.3. Expression of Mutant Enzyme Alleles in BCG and Assays of Enzyme Activity

The mutant allele encoding ∆D50∆E327 GlnA1 (numbering per homologous enzyme in *Salmonella typhimurium* [30] but actually representing ∆D54∆E335 deletions in *M. tuberculosis* GlnA1) was ligated into the shuttle plasmid pHV203 and electroporated into 3dBCG to yield 4dBCG. Immunoblotting was used to compare enzyme quantity. Bacterial lysates were prepared from 25 mL of 10^8^ cfu/mL of each strain grown for 48 h in Middlebrook 7H9 media with 10% oleic acid-dextrose-catalase (OADC) enrichment supplemented with 0.2% glycerol and 0.05% Tween80. Lysates were adjusted to a standard A280 value, applied to a SDS-12% PAGE gel for separation of proteins by electrophoresis and transferred to nitrocellulose membranes and hybridized with a 1:20 dilution of the anti-GlnA1 antibody IT-58 was obtained from Colorado State University as part of NIH, NIAID Contract HHSN26620040091C, “Tuberculosis Vaccine Testing and Research Materials.” To measure glutamine synthetase activity we monitored γ-glutamylhydroxamate formation spectrophotometrically at 540 nm by the γ-glutamyl transfer reaction [90]. As a factor in the cell lysate partially inhibited the assay, we first treated the lysate with 50% ammonium sulfate and then dialyzed the soluble portion with assay buffer, 0.04 M potassium phosphate, pH 7.0, and concentrated to 1 mL before performing the assay. Hydroxylamine, glutamine, and gamma-glutamylhydroxamate were purchased from Sigma.

### 3.4. Animal Procedures

Experiments involving the monitoring of the vaccine strains in the organs of mice were approved by the Vanderbilt Institutional Animal Care and Use Committee (Protocol No. M/06/069). Experiments involving vaccination-challenge were approved by the Syracuse VAMC Subcommittee on Animal Studies (ACORP Protocol No. 005). Female C57BL/6 mice aged 5–6 weeks were purchased from Jackson Laboratories (Bar Harbor, ME, USA). Methods for preparing inocula of the vaccines and challenge strain are described above. Bacterial suspensions were adjusted to a standard absorbance and diluted to achieve the desired inoculum. In studies to compare the clearance of vaccine strains, mice were inoculated by tail vein or retro-orbital injection. 

In the vaccination-challenge experiments mice were vaccinated subcutaneously and rested until challenge by aerosol with 100 cfu of Erdman S-1. For aerosol infection with Erdman S-1, the organism, supplied frozen, was removed from the freezer, thawed, sonicated and diluted to a final concentration of 1 × 10^6^ cfu/mL and ten mL of the inoculum was aerosolized to deliver ~100 cfu/mouse. Some mice were euthanized and their lungs harvested 24 h post-challenge to verify the challenge dose.

To enumerate bacteria in the organs of mice, mice were euthanized with CO_2_ inhalation and the spleens and left lungs were removed aseptically and placed in a sealed grinding assembly (IdeaWorks! Laboratory Products, Syracuse, NY, USA) attached to a Glas-Col Homogenizer (Terre Haute, IN, USA). Viable bacterial counts were determined by titration on 7H10 agar plates containing 10% OADC supplemented with 2 μg/mL of 2-thiophene carbonylhydrazide. During the course of the 26-week vaccination-challenge experiment one mouse in the 4dBCG vaccination arm was euthanized for humane reasons related to problems with excessive grooming.

### 3.5. Histopathology

At the time of harvest, right lungs from each vaccination arm were submersed in formalin. The lungs from each mouse were routinely cut at two levels in antero-posterior sagittal planes. All pieces from a single mouse were embedded flat on the cut surfaces in a single paraffin block. The lungs were cut into 4-μm sections and stained with hematoxylin and eosin (H&E). Occasionally the sections displayed the whole sagittal plane of the right lung at different levels. To illustrate differences in the magnitude of parenchymal lung involvement and loss of alveolar architecture between different vaccination arms, the slides were laid on white paper and photographed with a digital camera. Then three photomicrographs covering 70 to 80% of the visible lung on the slide from three mice in each group were taken using a ×2 microscope objective and digital microscope camera (Olympus America, Inc., Melville, NY, USA). Higher-magnification views were taken as needed to display specific features. To prepare the multi-panel figures, we used Adobe^®^ Illustrator (Adobe Systems, Inc., San Jose, CA, USA) to place photographs within each figure. Then the composite figure was modified within Adobe^®^ Photoshop where autolevel, brightness, and contrast adjustments were applied uniformly to the whole figure to optimize the display.

### 3.6. Statistical Comparisons

Statistical analyses were performed using Prism 5.0 software (GraphPad) and only significant (*p* < 0.05) or close to significant (*p* < 0.1) values are indicated. Unless otherwise indicated, calculations were performed using the two-tailed Mann-Whitney test.

## 4. Conclusions

The strategy of preferring BCG daughter strains based on *in vivo* virulence and persistence has failed. The BCG vaccines designated as “strong” on the basis of historical criteria have problems with inadequate safety in immune suppressed persons, adverse reactions in normal hosts, poor memory T-cell responses, and poor protection against pulmonary TB. In this investigation we demonstrate that by targeting microbial enzymes implicated in immune suppression, the *in vivo* persistence of BCG is reduced while protection in a vaccine-challenge model is enhanced. We believe that the strategy of improving BCG by reducing its immune suppressive capacity addresses the major limitations of current BCG vaccines against TB, modifying BCG in a manner that should make it a safer and more effective vaccine against pulmonary tuberculosis in man. This strategy for improving BCG should also work well in combination with new boosting vaccines against TB, which are more likely to be effective if the initial priming vaccine is highly immunogenic rather than immune suppressive.
